# Understanding adolescent and young adult use of family physician services: a cross-sectional analysis of the Canadian Community Health Survey

**DOI:** 10.1186/1471-2296-12-118

**Published:** 2011-11-01

**Authors:** Bridget L Ryan, Moira Stewart, M Karen Campbell, John Koval, Amardeep Thind

**Affiliations:** 1 Post-Doctoral Fellow, Suite 245 - 100 Collip Circle, Centre for Studies in Family Medicine, The University of Western Ontario, London, Ontario, N6G 4X8 Canada; 2 Director, Suite 245 - 100 Collip Circle, Centre for Studies in Family Medicine, The University of Western Ontario, London, Ontario, N6G 4X8 Canada; 3 Chair, K201B Kresge Building, Department of Epidemiology and Biostatistics, The University of Western Ontario, London, Ontario, N6A 5C1 Canada; 4 Professor, K3D Kresge Building, Department of Epidemiology and Biostatistics, The University of Western Ontario, London, N6A 5C1 Ontario, Canada; 5 Associate Professor, Department of Epidemiology and Biostatistics Suite 245 - 100 Collip Circle, Centre for Studies in Family Medicine, The University of Western Ontario, London, Ontario, N6G 4X8 Canada; 6 Department of Family Medicine, Suite 245 - 100 Collip Circle, Centre for Studies in Family Medicine, The University of Western Ontario, London, Ontario, N6G 4X8 Canada

## Abstract

**Background:**

Primary health care is known to have positive effects on population health and may reduce at-risk behavior and health problems in adolescence. Yet little is known about the factors that are associated with adolescent and young adult utilization of family physician services. It is critical to determine the factors associated with utilization to inform effective primary health care policy. We address this gap in the primary health care literature by examining three issues concerning adolescent and young adult family physician use: inequity; the unique developmental stage of adolescence; and the distinction between utilization (users versus non-users) and intensity (high users versus low users).

**Methods:**

We conducted nested logistic regressions for two outcomes: utilization and intensity of family physician services for early adolescence, middle adolescence, and young adulthood using the 2005 Canadian Community Health Survey.

**Results:**

Chronic conditions were associated with utilization in early and middle adolescence and intensity in all age groups. Respondents from Quebec had lower odds of utilization. Those without a regular medical doctor had much lower odds of being users. The factors associated with use in early and middle adolescence were in keeping with parental involvement while the factors in young adulthood show the emerging independence of this group.

**Conclusions:**

We highlight key messages not known previously for adolescent and young adult use of family physician services. There is inequity concerning regional variation and for those who do not have a regular medical doctor. There is variation in factors associated with family physician services across the three age groups of adolescence. Health care and health care policies aimed at younger adolescents must consider that parents are still the primary decision-maker while older adolescents are more autonomous. There is variation in the factors associated with the two outcomes of utilization and intensity of services. Factors associated with utilization must be understood when considering the equitability of access to primary health care while factors associated with intensity must be understood when considering appropriate use of resources. The understanding gained from this study can inform health care policy that is responsive to the critical developmental stage of adolescence and young adulthood.

## Background

Primary health care is known to have positive effects on population health [1] and may reduce at-risk behavior and health problems in adolescence [2]. Yet little is known about the factors that are associated with adolescent and young adult use of family physician services. It is critical to determine the factors associated with use in order to inform effective primary health care policy. This paper addresses this gap in primary health care services research by examining three policy-relevant issues concerning adolescent and young adult use of family physician services: inequity; the unique developmental stage of adolescence; and the distinction between utilization (users versus non-users) and intensity (high users versus low users).

First, the issue of inequity was explored by examining the relationship of access to a regular medical doctor, geographic location, household income, and need, to family physician use. There are studies that look at adolescents' use of all types of physicians: United States [3-18], Canada [19,20], and Spain [21]. Many of these are solely descriptive, providing only mean number of visits or proportions of adolescents making visits, sometimes stratifying by age, race, income/insurance. Three of these examined family physician services [8,16,19]. None have information about whether adolescents had access to a regular medical doctor, a factor associated with use in adults [22,23] and important with the current shortage of family physicians, not only in Canada but in many parts of the world [24-30].

Second, the important developmental differences throughout adolescence and young adulthood were examined. Adolescent and young adult health care is distinct from adult care in that the responsibility for accessing and making decisions concerning care shifts from the parent in younger adolescence to the young adult. Yet often adolescents and young adults are included with children or with adults in research studies [9,11,21,31]. For example, in adulthood, females make more visits than males [31,32] and a Canadian study of adolescents 12 to 19 years, found that females made more visits for all types of physicians [20]. This study examined if there were sex differences for family physician utilization and whether this varied depending on the age of the adolescent.

Third, the distinction was made between users and non-users (utilization), and among users, between high users and low users (intensity). Health care use has usually been modeled as a count outcome, or as a dichotomy between those who used services and those who did not. Both these classifications assume that the factors associated with use will be the same for non-users, low volume users and high volume users. In fact, there may be factors that distinguish users from non-users, while other factors distinguish high users from low users. Two recent Canadian studies conceptualized adult health care service use as a two level model [31,32]. However, no adolescent studies were found that conceptualize physician use in this way.

Health care policy and planning should be informed by information that takes into account these important distinctions in adolescent and young adult health care.

## Methods

A secondary analysis was conducted using the 2005 Canadian Community Health Survey (CCHS)[33]. The CCHS is a multi-stage stratified cluster design, population-based, cross-sectional health survey administered to Canadians 12 years of age and older. For this study, only adolescents and young adults (ages 12 to 24 years) were included. Details regarding the CCHS can be found on the Statistics Canada website [34]. Permission was received from the Statistics Canada Research Data Centre (RDC) to access these data at The University of Western Ontario. Approval from The University of Western Ontario Health Sciences Research Ethics Board was not required because this was a secondary analysis with no possibility of identification of individual survey respondents.

**Figure 1:**
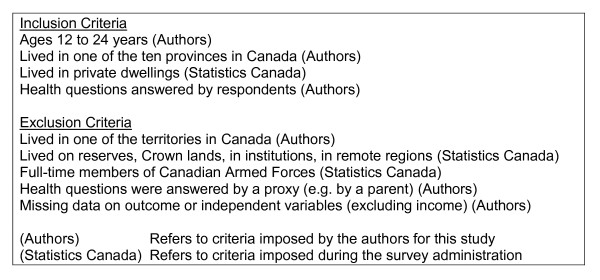
**Inclusion and exclusion criteria**.

Figure 1 summarizes the inclusion and exclusion criteria. The sample sizes for the analysis were: 12 to 14 year olds - 4985, 15 to 19 year olds - 8718, and 20 to 24 year olds - 6681. Cases included in the analysis were compared to those excluded because of missing data to determine if they varied by age, sex, and province of residence. In early adolescence, there was no difference among the provinces but females and younger early adolescents were more likely to have missing data. In middle adolescence, there was no difference between males and females or among ages but western provinces had more missing cases than eastern provinces. In young adulthood, there were no differences in the amount of missing data for any of sex, age, or province.

### Variables

The outcome, family physician use over the past year, was categorized as three levels: non-use (0 visits), low use (1 - 3 visits), and high use (4 or more visits). The two comparisons made were: 1) Utilization (users with non-users); and 2) Intensity of utilization within users (high users with low users).

Independent variables were chosen following Andersen's Behavioral Model of Health Services Use [35]. Wherever possible, the same variables were used for each of the three age groups to facilitate comparison across groups. Predisposing variables available and used were: age, sex, school attendance and educational attainment (highest level of education attained for middle adolescents and young adults), ethnicity (country of birth and racial origin), community belonging, marital status (young adults), and work status (middle adolescents and young adults). Enabling variables used were: household income adequacy (income level and household size combined), living arrangement (young adults), family physician access ("Do you have a regular medical doctor?"), and geography (urban or rural). Perceived need variables were: self-perceived general health, self-perceived mental health, opinion of own weight, and stress (available for middle adolescents and young adults only). Evaluated need variables were: BMI category, and the number of chronic conditions. Health practice variables used were: physical activity, smoking, sexual activity (available for middle adolescents and young adults only), and alcohol drinking. The CCHS does not provide health care system or external environment variables; however, province was used as a measure of context because health care in Canada is administered primarily at the provincial level.

### Analysis

Analyses were completed using Stata 10.0 for Windows [36]. Separate analyses were conducted for each of three groups, coinciding with the three developmental stages of adolescence [37]. This study complied with Statistics Canada requirements that data released from their Research Data Centres be weighted. Bootstrap weights provided by Statistics Canada were used to calculate variance estimates.

Descriptive statistics were used to examine the distributions of all variables within each of the three age groups for all respondents who answered the outcome question. Bivariate analyses were conducted to examine the association between all independent variables and family physician utilization and intensity of utilization. The variance inflation factor (VIF) was calculated for each independent variable and a VIF less than 10 was considered within acceptable limits for collinearity.

The multivariate analysis employed a nested logistic regression approach and used all cases with complete data. The first logistic regression addressed utilization (users versus non-users) and the second logistic regression, nested within users, examined intensity (high users versus low users). Thus a total of six regressions were generated, one for each outcome (utilization and intensity), for each of the three age groups. Within the logistic regression for intensity of utilization, an inclusive value (IV) parameter connecting the two outcomes was calculated. Because of the nesting of low use and high use within use, the three levels of outcome were not independent. Usual logistic regression models assume independence of alternative outcomes and similar error term distributions. Therefore a nested logistic regression model was employed where the IV parameter accounts for the covariance between the error terms. For each of the six logistic regressions, the main effects model was run first. The literature was reviewed for possible evidence of interactions but little was found. However, because there is inconsistent literature about the relationship between sex and utilization, where sex was significant in the main effects model, interaction terms were considered for sex with other demographic variables (education, income, ethnicity). Interactions were also considered between sex and sexual behavior variables (birth control, number of sexual partners) because females may have more need for family physician services for reproductive health issues (e.g. birth control prescriptions, cervical screening) than males. Regression diagnostics were conducted for each of the regressions using SAS Version 9.1 [38] examining the C statistics and the DFbeta statistics. No observations were found to be unduly influential and therefore all observations were retained in the final analyses.

Predicted probabilities were calculated comparing use and non-use for each of the three age groups by sex and number of chronic conditions. The other independent variables were held at their reference values for categorical variables and at their means for continuous variables. This calculates the probability that a "typical" adolescent or young adult would be a user. These results are consistent with those of the odds ratios from the logistic regressions but they can be more directly interpreted.

## Results

### Descriptive results

Table 1 describes the distribution of the outcome for each of the three age groups. Additional file 1.pdf describes the distribution of the independent variables for each age group.

**Table 1:** Distribution of family physician utilization by age groups

	Early adolescents	Middle adolescents	Young adults
	n = 5753	n = 9649	n = 7506
**Non-users (%)**	32.9	30.2	27.9
**Low users - 1 to 3 visits (%)**	52.6	50.8	50.4
**High users - 4 or more visits (%)**	14.5	19.0	21.7
**Total respondents (%)**	100.0	100.0	100.0
**Mean number of visits (standard deviation)**	1.9 (2.9)	2.4 (4.4)	2.8 (5.6)
**Median number of visits**	1	1	2
**Interquartile range number of visits**	0 - 2	0 - 3	0 - 3

### Nested logistic regression results

Tables 2 and 3 report the results of the multivariate logistic regressions for each age group for the two outcomes of utilization and intensity of utilization respectively. Additional file 2.pdf and Additional file 3.pdf provide the unadjusted odds ratios for the two outcomes for each age group.

**Table 2:** Logistic regressions for family physician utilization (use versus no use)

Stage of adolescence	Early			Middle			Young adult		
Sample size	4985			8718			6681		
Variable (reference)	OR^a^	CI-L^a^	CI-U^a^	OR^a^	CI-L^a^	CI-U^a^	OR^a^	CI-L^a^	CI-U^a^
**CONTEXT**									
Province (Ontario)									
*Atlantic*	0.86	0.64	1.17	1.05	0.83	1.34	0.92	0.71	1.19
*Quebec*	**0.53**	**0.37**	**0.77**	**0.70**	**0.55**	**0.88**	0.95	0.73	1.23
*Manitoba*	0.77	0.51	1.17	1.20	0.81	1.77	**1.51**	**1.00**	**2.27**
*Saskatchewan*	0.95	0.65	1.37	1.38	1.00	1.91	1.13	0.75	1.72
*Alberta*	1.26	0.89	1.76	1.24	0.93	1.64	**1.55**	**1.16**	**2.07**
*British Columbia*	0.92	0.70	1.23	1.04	0.83	1.31	0.99	0.74	1.32
**PREDISPOSING**									
Sex (Male)									
*Female*	1.10	0.91	1.34	**1.54**	**1.26**	**1.88**	**1.58**	**1.23**	**2.03**
Education attainment	-	-	-	**1.11**	**1.01**	**1.23**	1.02	0.93	1.12
Birth country (Canada)									
*Other *	1.28	0.86	1.92	0.86	0.65	1.15	0.93	0.64	1.34
Racial origin (White)									
*Visible minority*	**0.74**	**0.57**	**0.97**	1.24	0.99	1.54	0.98	0.72	1.34
Community belonging	1.10	0.96	1.27	1.09	1.00	1.20	**1.11**	**1.00**	**1.23**
**ENABLING**									
Household income (Middle)									
*Low income*	1.03	0.75	1.40	**0.76**	**0.59**	**0.97**	0.82	0.60	1.11
*Low-middle income*	1.07	0.80	1.44	0.83	0.64	1.07	0.94	0.69	1.27
*Low-high income*	1.01	0.74	1.39	0.92	0.72	1.17	1.14	0.86	1.52
*High income*	1.06	0.77	1.47	1.02	0.78	1.35	0.84	0.63	1.12
*Income missing*	1.01	0.76	1.35	0.82	0.66	1.03	0.76	0.57	1.02
Living arrangement (Unattached)									
*With spouse (and children)*	-	-	-	-	-	-	1.19	0.64	2.12
*With parent (and siblings)*	-	-	-	-	-	-	1.17	0.93	1.48
*Other (e.g. roommates)*	-	-	-	-	-	-	1.51	0.97	2.34
Regular medical doctor (Yes)									
*No*	**0.39**	**0.30**	**0.51**	**0.35**	**0.30**	**0.42**	**0.40**	**0.33**	**0.48**
Urban or rural (Urban)									
*Rural*	0.85	0.68	1.05	0.86	0.73	1.02	**0.79**	**0.64**	**0.98**
**NEED - PERCEIVED**									
Self-perceived health	0.98	0.86	1.11	1.00	0.91	1.10	0.93	0.81	1.06
Self-perceived mental health	0.97	0.82	1.13	**0.91**	**0.83**	**0.99**	0.93	0.83	1.05
Opinion of weight (About right)									
*Underweight*	**0.69**	**0.48**	**0.98**	1.15	0.86	1.55	1.11	0.76	1.63
*Overweight*	0.94	0.68	1.29	1.09	0.84	1.43	1.24	0.96	1.61
Stress	-	-	-	1.04	0.96	1.14	1.10	0.99	1.21
**NEED - EVALUATED**									
BMI (20-24 years) (Normal)									
*Underweight*	0.79	0.45	1.39	0.91	0.62	1.35	0.78	0.51	1.18
*At risk of overweight*	0.87	0.60	1.26	0.94	0.75	1.18	-	-	-
*Overweight*	1.14	0.75	1.72	0.96	0.70	1.31	0.88	0.70	1.10
*Obese*	-	-	-	-	-	-	0.81	0.57	1.60
Number of chronic conditions (None)									
*1 condition*	**1.62**	**1.27**	**2.07**	**1.23**	**1.03**	**1.46**	1.03	0.84	1.27
*2 conditions*	1.44	0.90	2.31	**1.36**	**1.09**	**1.71**	0.90	0.65	1.25
*3 conditions*	**2.48**	**1.17**	**5.26**	**2.18**	**1.53**	**3.10**	0.98	0.63	1.51
*4+ conditions*	1.61	0.36	7.26	**2.08**	**1.03**	**4.20**	0.72	0.33	1.55
**HEALTH PRACTICES**									
Physical activity (Inactive)									
*Active*	1.06	0.81	1.39	1.01	0.85	1.21	0.92	0.75	1.12
*Moderate*	0.80	0.60	1.06	1.03	0.86	1.24	1.01	0.82	1.26
Smoking (Never)									
*Daily(Ever for Early)*	1.16	0.81	1.68	0.83	0.64	1.07	**0.74**	**0.57**	**0.96**
*Occasional*	-	-	-	1.27	0.93	1.73	1.33	1.00	1.78
*Former*	-	-	-	0.92	0.75	1.13	0.88	0.71	1.08
Number of sexual partners	-	-	-	1.07	0.94	1.22	1.02	0.92	1.14
Alcohol frequency (No drinking)									
*Low frequency (Ever for Early)*	0.77	0.58	1.03	**1.24**	**1.01**	**1.52**	1.30	0.92	1.84
*High frequency*	-	-	-	1.33	0.98	1.81	1.40	0.96	2.05
Heavy drinking (No)									
*Yes*	1.35	0.87	2.11	1.06	0.86	1.29	1.11	0.89	1.39
**IV PARAMETER**	1.70	0.16	17.94	2.13	0.87	5.23	**4.85**	**2.02**	**11.64**

a - OR indicates odds ratios; CI-L and CI-U indicates lower and upper confidence intervals respectively; Bolded indicates significant results at p ≤ 0.05

' - ' in OR cell indicates variable was not applicable and therefore not used for the particular age group

Variables included but not significant in any regression: age, school attendance, marital status, work status, and birth control use

**Table 3:** Logistic regressions for family physician intensity of utilization (high use versus low use)

Stage of adolescence	Early			Middle			Young adult		
Sample size	3378			6237			4804		
Variable (reference)	OR^a^	CI-L^a^	CI-U^a^	OR^a^	CI-L^a^	CI-U^a^	OR^a^	CI-L^a^	CI-U^a^
**CONTEXT**									
Province (Ontario)									
*Atlantic*	1.07	0.75	1.52	**1.30**	**1.00**	**1.68**	1.08	0.79	1.46
*Quebec*	**0.57**	**0.38**	**0.86**	**0.51**	**0.38**	**0.68**	**0.53**	**0.40**	**0.71**
*Manitoba*	0.72	0.44	1.20	1.29	0.88	1.88	0.84	0.51	1.36
*Saskatchewan*	1.17	0.77	1.78	**1.74**	**1.28**	**2.36**	1.44	0.98	2.11
*Alberta*	1.10	0.74	1.63	**1.42**	**1.08**	**1.87**	0.98	0.72	1.32
*British Columbia*	1.11	0.79	1.55	**1.46**	**1.16**	**1.83**	1.30	0.95	1.78
**PREDISPOSING**									
Sex (Male)									
*Female*	0.91	0.72	1.15	1.23	0.97	1.56	**2.13**	**1.68**	**2.70**
Education attainment	-	-	-	0.95	0.84	1.08	1.11	1.00	1.25
Birth country (Canada)									
*Other *	1.16	0.73	1.84	**1.52**	**1.11**	**2.10**	1.01	0.70	1.47
Racial origin (White)									
*Visible minority*	0.88	0.64	1.23	0.94	0.72	1.22	0.91	0.67	1.24
Community belonging	0.93	0.77	1.12	1.04	0.94	1.15	1.11	0.98	1.25
**ENABLING**									
Household income (Middle)									
*Low income*	1.28	0.89	1.83	0.92	0.67	1.25	1.02	0.73	1.43
*Low-middle income*	0.83	0.57	1.20	0.99	0.74	1.32	1.04	0.75	1.45
*Low-high income*	0.78	0.53	1.15	0.85	0.63	1.16	0.94	0.67	1.30
*High income*	0.93	0.60	1.45	1.06	0.78	1.44	0.80	0.55	1.17
*Income missing*	1.18	0.79	1.75	1.04	0.80	1.36	0.86	0.60	1.23
Living arrangement (Unattached)									
*With spouse (and children)*	-	-	-	-	-	-	**1.77**	**1.11**	**2.82**
*With parent (and siblings)*	-	-	-	-	-	-	1.08	0.84	1.40
*Other (e.g. roommates)*	-	-	-	-	-	-	1.20	0.81	1.77
Regular medical doctor (Yes)									
*No*	1.01	0.61	1.67	0.85	0.64	1.13	0.91	0.70	1.20
Urban or rural (Urban)									
*Rural*	0.95	0.73	1.25	0.94	0.77	1.15	1.09	0.83	1.45
**NEED - PERCEIVED**									
Self-perceived health	0.96	0.80	1.14	**1.12**	**1.00**	**1.26**	**1.35**	**1.19**	**1.53**
Self-perceived mental health	**1.22**	**1.06**	**1.41**	1.10	0.99	1.21	**1.14**	**1.00**	**1.29**
Opinion of weight (About right)									
*Underweight*	1.10	0.68	1.78	1.09	0.79	1.50	0.99	0.63	1.56
*Overweight*	1.03	0.68	1.55	0.87	0.68	1.13	0.82	0.61	1.10
Stress	-	-	-	**1.14**	**1.03**	**1.26**	**1.15**	**1.02**	**1.30**
**NEED-EVALUATED**									
BMI (20-24 years) (Normal)									
*Underweight*	0.69	0.32	1.50	0.88	0.53	1.47	1.44	0.93	2.24
*At risk of overweight*	**1.67**	**1.19**	**2.35**	1.06	0.81	1.38	-	-	-
*Overweight*	1.49	0.92	2.40	1.34	0.94	1.92	1.11	0.83	1.49
*Obese*	-	-	-	-	-	-	**1.65**	**1.11**	**2.44**
Number of chronic conditions (None)									
*1 condition*	1.31	0.99	1.73	**1.49**	**1.21**	**1.84**	**1.46**	**1.14**	**1.87**
*2 conditions*	**1.92**	**1.41**	**2.61**	**1.68**	**1.32**	**2.14**	**2.45**	**1.83**	**3.27**
*3 conditions*	**2.67**	**1.71**	**4.18**	**2.19**	**1.62**	**2.96**	**2.66**	**1.86**	**3.80**
*4+ conditions*	**4.53**	**2.40**	**8.55**	**5.22**	**3.69**	**7.36**	**4.46**	**2.96**	**6.72**
**HEALTH PRACTICES**									
Physical activity (Inactive)									
*Active*	**1.35**	**1.01**	**1.82**	1.16	0.94	1.43	1.14	0.91	1.43
*Moderate*	1.16	0.82	1.64	0.95	0.75	1.20	1.02	0.80	1.29
Smoking (Never)									
*Daily (Ever for Early)*	1.25	0.78	2.00	1.12	0.85	1.48	0.96	0.73	1.26
*Occasional*	-	-	-	1.26	0.89	1.78	0.94	0.67	1.32
*Former*	-	-	-	**1.31**	**1.05**	**1.63**	1.01	0.78	1.31
Number of sexual partners	-	-	-	1.02	0.89	1.16	**1.18**	**1.05**	**1.34**
Alcohol frequency (No drinking)									
*Low frequency (Ever for Early)*	1.23	0.87	1.74	0.99	0.78	1.26	0.96	0.64	1.43
*High frequency*	-	-	-	0.79	0.57	1.12	0.90	0.59	1.38
Heavy drinking (No)									
*Yes*	1.18	0.65	2.15	**1.25**	**1.02**	**1.55**	0.92	0.70	1.22
**INTERACTIONS**									
Sex (Male) × Birth control (Not sexually active)									
*female birth control yes*				**1.81**	**1.26**	**2.60**			
*female birth control no*				**2.53**	**1.07**	**6.00**			

a - OR indicates odds ratios; CI-L and CI-U indicates lower and upper confidence intervals respectively; Bolded indicates significant results at p ≤ 0.05

' - ' in an OR cell indicates this variable was not applicable and therefore not used for the particular age group

Variables included but not significant in any regression: age, school attendance, marital status, work status, and birth control use

### Utilization of family physician (users versus non-users)

With respect to utilization, respondents from Quebec had lower odds of being users than those from Ontario for early and middle adolescents but this did not reach significance for young adults. Young adult respondents from Manitoba and Alberta had higher odds of being users than those from Ontario. Females had higher odds of being users in middle adolescence and young adulthood. Those early adolescents who indicated their racial origin as not white had lower odds of being users than those who indicated white. For young adults, an increasingly positive sense of community belonging had a significant association with being a user as did living in an urban area. Income was generally not associated with being a user except in the case of low income for middle adolescents. Respondents from all three age groups who did not have a regular medical doctor had lower odds of being users than those who had a regular medical doctor. Perceived need variables were associated with being a user for early adolescents where being underweight was associated with non-use, and for middle adolescents, where poorer self-perceived mental health was associated with non-use. The association of having chronic conditions with being a user varied across the three age groups. In early adolescents, having 1 and 3 chronic conditions was associated with being a user while every level of having chronic conditions was associated with being a user in middle adolescents. In young adults, having chronic conditions was not associated with being a user. There were no significant interactions for any of the age groups.

### Intensity of family physician utilization (high users versus low users)

With respect to intensity, the odds of being a high user were lower for those from Quebec for all three age groups. As well, for middle adolescents, respondents from the Atlantic Provinces, Saskatchewan, Alberta, and British Columbia had higher odds of being high users than those from Ontario. Sex was a factor in middle adolescence as an interaction with the use of birth control. No other interactions were significant for any of the age groups. Sex was a main effect in young adulthood where females had higher odds of being high users. In middle adolescence, those respondents who were born in a country other than Canada had higher odds of being high users than those born in Canada. There was no association between income and being a high user for any of the age groups. For all age groups, the odds of being a high user increased with having more chronic conditions. The number of perceived need variables associated with being a high user increased with each age group.

### Predicted probabilities of being a user

For all age groups, the predicted probability of being user when the adolescent had a regular medical doctor ranged from 70 to 90 percent. For those without a regular medical doctor, the predicted probabilities of being a user were generally 20% less, ranging from 50 to 70 percent. Consistently in middle adolescence and young adulthood, males had an approximately 10% lower probability of being a user than females, regardless of whether they had a regular medical doctor.

## Discussion

Three messages concerning adolescent and young adult use of family physician services, not known previously, are highlighted: 1) inequity concerning access and geography; 2) differences in each sub-group of adolescence and young adulthood; and 3) the important distinction between utilization (users versus non-users) and intensity (high users versus low users).

The first key message concerns the inequitability of family physician services. Two important factors concerning inequity were associated with the use of family physician services - having access to a regular medical doctor and geographic variation. Having a regular medical doctor was highly associated with being a user for all three age groups. This is consistent with the adult literature where having a usual source of care has been related to use of utilization [22,23]. Conventional wisdom suggests that access to a regular doctor is not important to adolescents because they use school services or health clinics. This finding confirms that access to a regular doctor is as important to adolescents and young adults as it is to adults. The difference in the probability of being a user of family physician services between those with a regular medical doctor and those without was approximately 20%. This difference has significant policy implications with respect to ensuring universal access to primary health care for adolescents and young adults. Also, despite a federal universal health care system, adolescents from Quebec were less likely to use family physician services. A study using the 2001 Canadian Community Health Survey also found variation in utilization by province for adults [32]. This regional variation deserves more attention. However, consistent with equitable access was the finding that the presence of an increasing number of chronic conditions was a strong factor associated with utilization for early and middle adolescence and for intensity of services for all three age groups. As well, household income was for the most part not associated with being a user. This is in contrast to four American studies that found income positively associated with utilization [4,6,7,11]. It is thought that, because of universal access to health care in Canada, income should not be a barrier to receiving care. This appears to be the case for family physician utilization in this dataset.

The second key message is that adolescents are not a homogeneous group. The factors associated with use in the early and middle adolescent groups were in keeping with parental involvement in health care decision-making. The factors associated with use in young adulthood show the emerging independence of this age group. For example, the number of self-perceived need variables associated with intensity of use increased across the age groups. Previous adolescent studies have found a relationship between poorer health status and increased utilization [5,11,20,21]. In the current study, the relationship was not as straight-forward highlighting crucial differences among the three stages of adolescence. Additionally, females were higher users of family physician services starting in middle adolescence and the difference was not totally attributable to contraceptive needs. Health care policy must be sensitive to these developmental distinctions in order to be effective.

The third key message embedded in all the findings is that the factors associated with family physician utilization (users versus non-users) were different than those associated with intensity of use (high users versus low users). Future research must consider this important distinction when modeling health care use. This finding also suggests the necessity to be clear with respect to which outcome (utilization or intensity) health care policy is intended to address. Factors associated with utilization must be understood when considering universal equitable access to primary health care while factors associated with intensity must be understood when considering appropriate use of primary health care resources.

The clearest policy-relevant message that arises out of these findings is the distinctly different stages of adolescence. This can be seen when examining health care policy designed to encourage adoption of vaccinations for sexually transmitted infections (STIs). While efforts to encourage vaccination may successfully be directed to young adults, policies aimed at younger adolescents must consider that parents are still the primary decision-maker for younger adolescents. One study found that parental intent to vaccinate against STIs was significantly associated with adolescents' intent to accept STI vaccination [39]. The controversy that surrounded the uptake of the HPV vaccine was due partly to a lack of understanding regarding the role parents play in young adolescent health care which resulted in not addressing parental concerns about safety and parental autonomy [40].

This study employed the CCHS, a Canada-wide population-based study making it representative of most of the adolescent population. Because it is not possible to measure temporality in a cross-sectional study, it was not possible to determine causal relationships but rather only to report associations between the outcomes and the independent variables. However, many of the variables were not time sensitive; either they did not change (e.g. sex, country of birth) or they were stable over time (self-perceived health). The concern about the cross-sectional nature of the survey is somewhat balanced by the consistency of these variables over time. The variables most sensitive to change over time were the health practices.

Another limitation of the study may be the use of self-report measures. Some studies have found under-reporting of health practice in adolescence while others have not found this to be a large problem [41-43]. The inconsistent relationship of health practices with utilization may be, in part, due to variables being cross-sectional and based on self-report.

Future research could examine the factors associated with family physician use for specific groups of adolescents and young adults such as those with chronic conditions. Multi-level methods could be employed to examine contextual factors that might elaborate the variation found between geographic areas.

## Conclusions

There is limited primary health care services research that examines adolescent and young adult health care use employing multivariate analysis. This understanding of adolescent patterns of utilization, in addition to simply describing them, can inform health care policy that is responsive to this critical developmental stage.

## Competing interests

The authors declare that they have no competing interests.

## Authors' contributions

BLR designed the study, conducted the analysis, interpreted the results and wrote the manuscript. The remaining authors were part of BLR's Ph.D. thesis advisory committee. MS participated in the design of the study, assisted with the interpretation and helped draft the manuscript. MKC assisted in the design of the study and participated in drafting the analysis plan. JK assisted with statistical analysis especially with respect to the regression diagnostics. AT assisted with the statistical analysis especially with respect to the nested logistic regressions. All authors read and approved the final manuscript.

## Pre-publication history

The pre-publication history for this paper can be accessed here:

http://www.biomedcentral.com/1471-2296/12/118/prepub

## Supplementary Material

Additional file 1 **"Description of independent variables by age group"**. Click here for file

Additional file 2 **"Unadjusted logistic regressions for family physician utilization (use versus no use)"**. Click here for file

Additional file 3 **"Unadjusted logistic regressions for family physician intensity of utilization (high use versus low use)"**. Click here for file

## References

[B1] Starfield B Shi L Macinko J Contribution of primary care to health systems and health Milbank Q 2005 83 457 502 10.1111/j.1468-0009.2005.00409.x 16202000 PMC2690145

[B2] Klein D Short report: adolescents' health. Does having a family physician make a difference? Can Fam Physician 2003 49 1000 1002 12943359 PMC2214272

[B3] Ziv A Boulet JR Slap GB Utilization of physician offices by adolescents in the United States Pediatrics 1999 104 35 42 10.1542/peds.104.1.35 10390257

[B4] Klein JD Wilson KM McNulty M Kapphahn C Collins KS Access to medical care for adolescents: results from the 1997 Commonwealth Fund Survey of the Health of Adolescent Girls J Adolesc Health 1999 25 120 130 10.1016/S1054-139X(98)00146-3 10447039

[B5] Ryan S Riley A Kang M Starfield B The effects of regular source of care and health need on medical care use among rural adolescents Arch Pediatr Adolesc Med 2001 155 184 190 11177095 10.1001/archpedi.155.2.184

[B6] Goodman E Huang B Socioeconomic status, depression, and health service utilization among adolescent women Womens Health Issues 2001 11 416 426 10.1016/S1049-3867(01)00077-9 11566284

[B7] Yu SM Bellamy HA Schwalberg RH Drum MA Factors associated with use of preventive dental and health services among U.S. adolescents J Adolesc Health 2001 29 395 405 10.1016/S1054-139X(01)00252-X 11728889

[B8] Marcell AV Klein JD Fischer I Allan MJ Kokotailo PK Male adolescent use of health care services: where are the boys? J Adolesc Health 2002 30 35 43 10.1016/S1054-139X(01)00319-6 11755799

[B9] Elster A Jarosik J VanGeest J Fleming M Racial and ethnic disparities in health care for adolescents: a systematic review of the literature Arch Pediatr Adolesc Med 2003 157 867 874 10.1001/archpedi.157.9.867 12963591

[B10] Probst JC Moore CG Baxley EG Update: health insurance and utilization of care among rural adolescents J Rural Health 2005 21 279 287 10.1111/j.1748-0361.2005.tb00096.x 16294649

[B11] Simpson L Owens PL Zodet MW Chevarley FM Dougherty D Elixhauser A Health care for children and youth in the United States: annual report on patterns of coverage, utilization, quality, and expenditures by income Ambul Pediatr 2005 5 6 44 10.1367/A04-119R.1 15656707

[B12] Rand CM Shone LP Albertin C Auinger P Klein JD Szilagyi PG National health care visit patterns of adolescents: implications for delivery of new adolescent vaccines Arch Pediatr Adolesc Med 2007 161 252 259 10.1001/archpedi.161.3.252 17339506

[B13] Park MJ Paul MT Adams SH Brindis CD Irwin CE Jr The health status of young adults in the United States J Adolesc Health 2006 39 305 317 10.1016/j.jadohealth.2006.04.017 16919791

[B14] Fortuna RJ Robbins BW Halterman JS Ambulatory care among young adults in the United States Ann Intern Med 2009 151 379 385 19755363 10.7326/0003-4819-151-6-200909150-00002

[B15] Irwin CE Jr Adams SH Park MJ Newacheck PW Preventive care for adolescents: few get visits and fewer get services Pediatrics 2009 123 e565 e572 10.1542/peds.2008-2601 19336348

[B16] Nordin JD Solberg LI Parker ED Adolescent primary care visit patterns Ann Fam Med 2010 8 511 516 10.1370/afm.1188 21060121 PMC2975686

[B17] Hoover KW Tao G Berman S Kent CK Utilization of health services in physician offices and outpatient clinics by adolescents and young women in the United States: implications for improving access to reproductive health services J Adolesc Health 2010 46 324 330 10.1016/j.jadohealth.2009.09.002 20307820

[B18] Dempsey AF Freed GL Health care utilization by adolescents on medicaid: implications for delivering vaccines Pediatrics 2010 125 43 49 10.1542/peds.2009-1044 19948567

[B19] Brownell M Kozyrkyj A Roos N Friesen D Mayer T Sullivan K Health service utilization by Manitoba children Can J Public Health 2002 93 Suppl 2 S57 S62 12580392 10.1007/BF03403620 PMC6979766

[B20] Vingilis E Wade T Seeley J Predictors of adolescent health care utilization J Adolesc 2007 30 773 800 10.1016/j.adolescence.2006.10.001 17141307

[B21] Berra S Borrell C Rajmil L Estrada MD Rodriguez M Riley AW Perceived health status and use of healthcare services among children and adolescents Eur J Public Health 2006 16 405 414 10.1093/eurpub/ckl055 16644926

[B22] Brown ER Davidson PL Yu H Wyn R Andersen RM Becerra L Effects of community factors on access to ambulatory care for lower-income adults in large urban communities Inquiry 2004 41 39 56 10.5034/inquiryjrnl_41.1.39 15224959

[B23] Rust G Fryer GE Jr Phillips RL Jr Daniels E Strothers H Satcher D Modifiable determinants of healthcare utilization within the African-American population J Natl Med Assoc 2004 96 1169 1177 15481745 PMC2568455

[B24] Lakhan SE Laird C Addressing the primary care physician shortage in an evolving medical workforce Int Arch Med 2009 2 14 10.1186/1755-7682-2-14 19416533 PMC2686687

[B25] Sheldon GF Ricketts TC Charles A King J Fraher EP Meyer A The global health workforce shortage: role of surgeons and other providers Adv Surg 2008 42 63 85 18953810 10.1016/j.yasu.2008.04.006

[B26] Cho KH Roh YK Primary care physicians shortage: a Korean example Public Health Rev 2003 31 133 148 15255161

[B27] Roick C Heider D Gunther OH Kurstein B Riedel-Heller SG Konig HH Factors Influencing the Decision to Establish a Primary Care Practice: Results from a Postal Survey of Young Physicians in Germany Gesundheitswesen 2010 10.1055/s-0030-1268448 21161878

[B28] Joyce CM McNeil JJ Stoelwinder JU More doctors, but not enough: Australian medical workforce supply 2001-2012 Med J Aust 2006 184 441 446 16646743 10.5694/j.1326-5377.2006.tb00315.x

[B29] Joyce CM McNeil JJ Fewer medical graduates are choosing general practice: a comparison of four cohorts, 1980-1995 Med J Aust 2006 185 102 104 16842069 10.5694/j.1326-5377.2006.tb00484.x

[B30] Toker A Shvarts S Glick S Reuveni H A report card on the physician work force: Israeli health care market--past experience and future prospects Health Policy 2010 97 38 43 10.1016/j.healthpol.2010.03.004 20399526

[B31] Nabalamba A Millar WJ Going to the doctor Health Reports 2007 18 23 35 17441441

[B32] Asada Y Kephart G Equity in health services use and intensity of use in Canada BMC Health Serv Res 2007 7 41 10.1186/1472-6963-7-41 17349059 PMC1829158

[B33] Statistics Canada Canadian Community Health Survey (CCHS) Cycle 3.1 (2005) Questionnaire 2004 http://www.statcan.gc.ca/concepts/health-sante/cycle3_1/q-eng.htm

[B34] Statistics Canada Canadian Community Health Survey (CCHS) Cycle 3.1 (2005) User's Guide 2005 http://www.statcan.gc.ca/cgi-bin/imdb/p2SV.pl?Function=getDocumentation&Item_Id=46559&lang=en&db=imdb&adm=8&dis=2

[B35] Andersen RM Revisiting the behavioral model and access to medical care: does it matter? J Health Soc Behav 1995 36 1 10 10.2307/2137284 7738325

[B36] Stata Corp LP Stata/SE 10.0 for Windows. 2008 2008 College Station, TX, Stata Corp. LP

[B37] Irwin CE Jr Burg SJ Uhler CC America's adolescents: where have we been, where are we going? J Adolesc Health 2002 31 91 121 10.1016/S1054-139X(02)00489-5 12470908

[B38] SAS Institute Inc. SAS/Stat 9.1. 2009 2009 Cary, NC, SAS Institute

[B39] Sturm LA Mays RM Zimet GD Parental beliefs and decision making about child and adolescent immunization: from polio to sexually transmitted infections J Dev Behav Pediatr 2005 26 441 452 10.1097/00004703-200512000-00009 16344662

[B40] Flogging gardasil Nat Biotechnol 2007 25 261 17344862 10.1038/nbt0307-261

[B41] Brener ND Billy JOG Grady WR Assessment of factors affecting the validity of self-reported health-risk behavior among adolescents: Evidence from the scientific literature Journal of Adolescent Health 2003 33 436 457 10.1016/S1054-139X(03)00052-1 14642706

[B42] Caraballo RS Giovino GA Pechacek TF Self-reported cigarette smoking vs. serum cotinine among US adolescents Nicotine & Tobacco Research 2004 6 19 + 10.1080/14622200310001656821 14982684

[B43] Sieving R Hellerstedt W McNeely C Fee R Snyder J Resnick M Reliability of self-reported contraceptive use and sexual behaviors among adolescent girls Journal of Sex Research 2005 42 159 166 10.1080/00224490509552269 16123846

