# Supplementary material for: Understanding adolescent and young adult use of family physician services: a cross-sectional analysis of the Canadian Community Health Survey
Source: BMC Fam Pract. 2011 Nov 1;12:118. doi: 10.1186/1471-2296-12-118 (PMC3219741; doi:10.1186/1471-2296-12-118)
Supplement: Additional file 1 — "Description of independent variables by age group". [file 1471-2296-12-118-S1.PDF]

Additional file 1 – Description of independent variables by age group

Independent variables	Values (*Reference)	Early adolescents n = 5753 %	Middle adolescents n = 9649 %	Young adults n = 7506 %
**CONTEXT**				
**Province**	Ontario*	38.80	40.41	39.82
	Atlantic	7.32	7.29	6.70
	Quebec	22.56	22.30	22.86
	Manitoba	3.74	3.61	3.29
	Saskatchewan	3.22	3.24	3.02
	Alberta	12.36	9.93	10.66
	British Columbia	11.99	13.21	13.66
	Missing	0.00	0.00	0.00
**PREDISPOSING**				
Sex	Male*	52.22	50.12	52.01
	Female	47.78	49.88	47.99
	Missing	0.00	0.00	0.00
Education – School attendance	Full-time*	n/a	75.50	38.60
	Part-time	n/a	3.95	7.07
	Not attending	n/a	18.67	52.41
	Missing	n/a	1.89	1.92
Education attainment	Less than secondary	n/a	59.04	7.85
	Secondary grad	n/a	15.86	21.77
	Other post-secondary	n/a	17.95	24.49
	Post-secondary grad	n/a	5.05	43.73
	Missing	n/a	2.09	2.17
Ethnicity -birth country	Canada*	90.04	85.06	83.01
	Other	8.90	13.20	15.19
	Missing	1.06	1.74	1.80
Ethnicity -racial origin	White*	78.55	76.93	75.38
	Other	19.57	20.84	22.56
	Missing	1.86	2.23	2.07
Social network - Community belonging	Very weak	3.23	7.92	12.48
	Somewhat weak	10.52	26.51	33.33
	Somewhat strong	57.48	51.25	43.11
	Very strong	27.12	13.78	10.48
	Missing	1.67	0.53	0.57
Marital status	Single*	n/a	n/a	82.24
	Common-law	n/a	n/a	11.64
	Married	n/a	n/a	6.09
	Missing	n/a	n/a	0.03

Work status	Full-time*	n/a	18.12	51.95
	Part-time	n/a	32.99	19.45
	Not working	n/a	46.55	26.22
	Missing	n/a	2.33	2.38
**ENABLING**				
Household income	Low	19.57	17.26	18.94
	Low-middle	19.00	14.74	15.83
	Middle*	18.13	15.13	15.67
	Middle-high	15.09	13.87	15.16
	High	10.55	10.83	13.31
	Missing (in analysis)	17.66	28.18	21.09
Living arrangement	Unattached*	n/a	n/a	22.58
	With spouse/children	n/a	n/a	15.27
	With parents/siblings	n/a	n/a	52.48
	Other	n/a	n/a	9.10
	Missing	n/a	n/a	0.57
Regular medical doctor	Yes*	86.77	83.42	75.30
	No	12.34	16.06	24.58
	Missing	0.89	0.52	0.13
Urban or rural location	Urban*	79.80	81.44	86.52
	Rural	20.20	18.56	13.48
	Missing	0.00	0.00	0.00
**NEED - PERCEIVED**				
General health	Excellent	25.15	22.59	24.54
	Very good	43.28	44.58	44.54
	Good	27.62	27.95	26.01
	Fair or poor	3.81	4.81	4.89
	Missing	0.14	0.06	0.04
Mental health	Excellent	40.34	41.50	39.77
	Very good	37.09	34.97	36.64
	Good	19.31	19.48	18.78
	Fair or poor	2.78	3.95	4.80
	Missing	0.47	0.07	0.01
Opinion of own weight	Just about right*	78.24	75.22	69.70
	Underweight	6.47	8.18	7.46
	Overweight	10.69	14.52	21.54
	Missing	4.61	2.08	1.29

Stress	Not at all stressful	n/a	9.90	7.97
	Not very stressful	n/a	28.87	25.50
	A bit stressful	n/a	45.55	44.68
	Quite a bit/extremely stressful	n/a	15.54	21.74
	Missing	n/a	0.16	0.09
**NEED - EVALUATED**				
BMI category	Healthy/normal*	67.30	75.50	61.87
	Underweight	2.62	4.19	5.52
	At risk of overweight	13.47	11.13	n/a
	Overweight	7.75	6.56	21.61
	Obese	n/a	n/a	7.95
	Missing	8.85	2.62	3.04
Number of chronic conditions	0*	52.23	49.86	43.90
	1	28.84	27.47	27.64
	2	13.05	13.45	15.69
	3	4.17	5.23	6.87
	4+	1.70	3.98	5.89
	Missing	0.00	0.00	0.00
**HEALTH PRACTICES**				
Physical activity	Active	52.41	46.64	36.78
	Moderate	22.72	22.07	24.23
	Inactive*	24.58	31.07	38.73
	Missing	0.28	0.22	0.25
Smoking	Never*	92.70	65.68	43.17
	Ever	6.87	n/a	n/a
	Daily	n/a	10.22	20.13
	Occasional	n/a	7.46	10.59
	Former	n/a	16.30	25.81
	Missing	0.43	0.35	0.31
Sexual activity - Number of partners	0	n/a	58.51	20.97
	1	n/a	25.01	50.95
	2	n/a	7.05	11.79
	3	n/a	2.70	5.54
	4+	n/a	2.89	6.29
	Missing	n/a	3.82	4.46
Sexual activity- Birth control	Yes	n/a	32.28	65.11
	No	n/a	5.15	9.17
	Not sexually active*	n/a	58.51	20.97
	Missing	n/a	4.05	4.74

Alcohol frequency	No drinking*	80.57	29.12	11.39
	Drinking	18.44	n/a	n/a
	<1/mo to 2-3/mo	n/a	49.51	42.23
	1/wk to 1/day	n/a	20.14	45.43
	Missing	0.99	1.23	0.96
Alcohol heavy drinking	No episodes*	94.51	52.50	31.19
	Episodes	4.47	46.14	67.51
	Missing	1.03	1.36	1.31

n/a - indicates this variable was either not collected for the particular age group or was not applicable for the developmental stage or there was little to no variation in the data.
