# Supplementary material for: Understanding adolescent and young adult use of family physician services: a cross-sectional analysis of the Canadian Community Health Survey
Source: BMC Fam Pract. 2011 Nov 1;12:118. doi: 10.1186/1471-2296-12-118 (PMC3219741; doi:10.1186/1471-2296-12-118)
Supplement: Additional file 2 — "Unadjusted logistic regressions for family physician utilization (use versus no use)". [file 1471-2296-12-118-S2.PDF]

Additional file 2: Unadjusted logistic regressions for family physician utilization (use versus no use)

**Stage of adolescence**	**Early**			**Middle**			**Young adult**		
**Sample size**	**4985**			**8718**			**6681**		
**Variables (reference)**	**OR[a]**	**CI-L[a]**	**CI-U[a]**	**OR[a]**	**CI-L[a]**	**CI-U[a]**	**OR[a]**	**CI-L[a]**	**CI-U[a]**
**CONTEXT**									
Province (Ontario)									
Atlantic	0.93	0.70	1.25	1.06	0.86	1.30	1.00	0.78	1.28
Quebec	**0.44**	**0.35**	**0.55**	**0.55**	**0.46**	**0.65**	**0.66**	**0.54**	**0.81**
Manitoba	**0.67**	**0.46**	**0.97**	1.03	0.72	1.48	1.22	0.84	1.77
Saskatchewan	0.92	0.64	1.33	**1.37**	**1.04**	**1.81**	1.04	0.73	1.47
Alberta	1.20	0.88	1.65	1.13	0.88	1.45	1.26	0.96	1.64
British Columbia	0.95	0.72	1.24	1.06	0.86	1.31	1.07	0.83	1.37
**PREDISPOSING**									
Age	0.94	0.85	1.03	1.05	0.96	1.10	1.04	0.98	1.09
Sex (Male)									
Female	1.08	0.91	1.28	**1.69**	**1.48**	**1.93**	**2.33**	**1.98**	**2.75**
Education attendance (Attending full-time)									
Attending part-time	-	-	-	1.11	0.80	1.54	1.26	0.89	1.79
Not attending	-	-	-	0.99	0.84	1.16	0.97	0.83	1.13
Education attainment	-	-	-	1.06	0.98	1.14	**1.10**	**1.02**	**1.18**
Birth country (Canada)									
Other	1.09	0.78	1.54	0.82	0.65	1.04	0.75	0.58	0.97
Racial origin (White)									
Visible minority	0.88	0.71	1.10	0.98	0.81	1.18	0.84	0.68	1.04
Community belonging	**1.16**	**1.02**	**1.32**	**1.13**	**1.04**	**1.22**	**1.16**	**1.06**	**1.26**
Marital status (Single)									
Common-law	-	-	-	-	-	-	1.14	0.91	1.41
Married	-	-	-	-	-	-	1.12	0.83	1.50
Work status (working full-time)									
Working part-time	-	-	-	1.11	0.93	1.33	1.23	0.99	1.53
Not working	-	-	-	0.96	0.81	1.14	0.96	0.80	1.16

**ENABLING**									
Household income (Middle)									
Low income	0.96	0.74	1.24	**0.69**	**0.55**	**0.87**	**0.76**	**0.58**	**0.99**
Low-middle income	1.00	0.76	1.31	**0.77**	**0.60**	**0.98**	0.94	0.71	1.23
Low-high income	1.03	0.78	1.37	0.91	0.72	1.16	1.17	0.89	1.53
High income	1.19	0.87	1.63	1.08	0.84	1.39	0.82	0.62	1.07
Income missing	1.03	0.78	1.34	0.86	0.69	1.06	**0.75**	**0.57**	**0.99**
Living arrangement (Unattached)									
With spouse (and children)	-	-	-	-	-	-	**1.47**	**1.20**	**1.79**
With parent (and siblings)	-	-	-	-	-	-	**1.20**	**1.00**	**1.44**
Other (e.g. roommates)	-	-	-	-	-	-	**1.44**	**1.02**	**2.01**
Regular medical doctor (Yes)									
No	**0.31**	**0.25**	**0.40**	**0.31**	**0.26**	**0.36**	**0.35**	**0.30**	**0.42**
Urban or rural (Urban)									
Rural	0.88	0.72	1.06	0.88	0.76	1.03	0.86	0.71	1.04
**NEED - PERCEIVED**									
Self-perceived health	0.99	0.88	1.10	**1.08**	**1.00**	**1.17**	1.05	0.96	1.16
Self-perceived mental health	1.01	0.90	1.13	1.02	0.94	1.10	1.01	0.92	1.11
Opinion of weight (About right)									
Underweight	0.73	0.52	1.02	1.00	0.78	1.29	0.90	0.66	1.21
Overweight	1.03	0.77	1.38	1.19	0.98	1.44	**1.26**	**1.05**	**1.51**
Stress	-	-	-	**1.16**	**1.08**	**1.25**	**1.22**	**1.12**	**1.33**
**NEED - EVALUATED**									
BMI (Normal)									
Underweight	0.68	0.42	1.11	0.81	0.56	1.17	1.12	0.79	1.58
At risk of overweight	0.97	0.76	1.23	0.92	0.76	1.12	-	-	-
Overweight	1.14	0.82	1.58	1.00	0.80	1.25	0.93	0.78	1.11
Obese	-	-	-	-	-	-	1.04	0.81	1.34

Number of chronic conditions (None)									
1 condition	**1.60**	**1.31**	**1.96**	**1.32**	**1.14**	**1.54**	**1.28**	**1.06**	**1.53**
2 conditions	**1.52**	**1.15**	**2.02**	**1.60**	**1.31**	**1.94**	**1.50**	**1.20**	**1.88**
3 conditions	**3.00**	**1.78**	**5.05**	**2.61**	**1.93**	**3.54**	**1.93**	**1.38**	**2.70**
4+ conditions	**2.19**	**1.10**	**4.36**	**3.93**	**2.71**	**5.70**	**2.47**	**1.54**	**3.96**
**HEALTH PRACTICES**									
Physical activity (Inactive)									
Active	1.22	0.98	1.51	1.06	0.92	1.23	1.00	0.85	1.18
Moderate	0.89	0.69	1.16	1.05	0.86	1.24	1.10	0.90	1.34
Smoking (Never)									
Daily (Ever for Early)	0.91	0.68	1.24	0.98	0.81	1.20	0.83	0.67	1.02
Occasional	-	-	-	**1.46**	**1.11**	**1.91**	**1.44**	**1.10**	**1.89**
Former	-	-	-	1.05	0.88	1.24	1.01	0.83	1.22
Number of sexual partners	-	-	-	**1.13**	**1.05**	**1.21**	1.07	0.99	1.16
Use birth control (Not sexually active)									
Yes	-	-	-	**1.30**	**1.13**	**1.49**	**1.39**	**1.13**	**1.71**
No	-	-	-	**1.46**	**1.05**	**2.03**	0.91	0.67	1.24
Alcohol frequency (No drinking)									
Low frequency (Ever for Early)	**0.78**	**0.63**	**0.96**	**1.34**	**1.15**	**1.56**	**1.64**	**1.24**	**2.17**
High frequency	-	-	-	**1.30**	**1.08**	**1.55**	**1.45**	**1.10**	**1.91**
Heavy drinking (No)									
Yes	1.06	0.73	1.54	**1.21**	**1.06**	**1.37**	1.10	0.92	1.32

a - OR indicates odds ratios; CI-L and CI-U indicates lower and upper confidence intervals respectively; Bolded indicates significant results at $p \leq 0.05$

' - ' in OR cell indicates variable was not applicable (or not available) and therefore not used for the particular age group
